# Initial Validation of a Chinese Version of the Mental Health Literacy Scale Among Chinese Teachers in Henan Province

**DOI:** 10.3389/fpsyt.2021.661903

**Published:** 2021-06-09

**Authors:** Shen Chen, Ke Chen, Shengnan Wang, Wei Wang, Yongxin Li

**Affiliations:** Department of Psychology, Institute of Psychology and Behavior, Henan University, Kaifeng, China

**Keywords:** mental health literacy, psychometric properties, reliability, validity, Chinese version of the Mental Health Literacy Scale

## Abstract

**Background:** Teachers' Mental health literacy (MHL) is one of the important factors that influence students' mental health literacy and even their mental health status. A valid, comprehensive measure is needed to adequately identify MHL levels in the Chinese teachers. Thus, this study aimed to validate a Chinese version of the Mental Health Literacy Scale (MHLS) among Chinese teachers.

**Methods:** A total of 367 Chinese primary and secondary school teachers in Henan province were enrolled to complete the Chinese version of MHLS and several validation measures.

**Results:** A parallel analysis supported a four-factor structure model of the Chinese version, but because of the low communalities and mean factor loadings, the univariate structure of the original scale was selected. Additionally, the criterion construct validity of the Chinese version was supported by significant correlations with self-efficacy in coping with mental health problems, mental health status, the stigma associated with receiving mental health treatment, and socially distancing from patients with mental illness. The Cronbach's α of the Chinese version was acceptable. Females, younger teachers, teachers with higher educational level, and full-time mental health teachers showed higher levels of MHL.

**Conclusion:** The Chinese version of MHLS is a valid and reliable tool to assess the level of Chinese teachers' MHL.

## Introduction

Mental health literacy (MHL), a multidimensional conception, was first defined by Jorm (1) as the “knowledge and beliefs about mental disorders which aid their recognition, management, or prevention.” He proposed seven attributes of MHL, “the ability to recognize specific disorders; knowing how to seek mental health information; knowledge of risk factors and causes, self-treatments, and professional help available; and attitudes that promote recognition and appropriate help-seeking” (1). In the ensuing years, Jorm (2) enlarged this concept and believed that MHL should not only refer to relevant theoretical knowledge, but also include content related to improving positive mental health outcomes. Kutcher et al. (3) extended the construct of MHL and emphasized the importance of fighting stigma, maintaining good mental health, and empowering a person to improve their help-seeking efficacy. Around the same time, the Canadian Alliance on Mental Illness and Mental Health argued that MHL should cover mental health promotion aspects as well as policy considerations (4). Although the connotation of MHL keeps evolving, the original definition proposed by Jorm is the most classic and comprehensive one (5).

MHL is derived from Health Literacy (HL), which means the ability to gain access to, understand, and use information to promote and maintain good health (6). Based on the understanding of HL, MHL is considered to be one of the important factors affecting mental health. A low level of MHL may be associated with high rates of suicides and adverse health consequences (7). It is also one of the prime factors that people with mental illnesses do not seek professional help (8). Some studies have found that people's MHL can be improved with interventions. A systematic review on the effectiveness of 27 school-based MHL programs illustrates their emerging evidence for knowledge improvement, attitudes, and help-seeking behavior amelioration (9). Kutcher and Wei (10) found that after participating in the training activities, the teachers' MHL improved significantly, simultaneously reducing the stigma related to mental illness notably. These studies help acknowledge the importance of MHL in improving mental health outcomes (4). Additionally, assessing the level of MHL accurately enables the development of educational programs aimed at promoting it (11), and the evaluation of the effectiveness of these programs. Therefore, several psychological instruments have been developed to measure the MHL of individuals.

Till date, according to the form of measurement, the existing measurement instruments can be roughly classified into two categories: vignette interview methods and scale-based measures. The vignette interview method, developed by Jorm et al. (1), is the first and widely used method of measurement (5). It presents a vignette of a person with one kind of mental disorder and then asks a series of questions relating to the participants' knowledge, attitude, and beliefs about mental disorders. The original version contained only two vignettes: depression and schizophrenia (1). However, six vignettes have now been developed for social phobia, depression with suicidal thoughts, early schizophrenia, chronic schizophrenia, substance abuse, and post-traumatic stress disorder (12). Later, other versions of the questionnaire were developed for teenagers and specific occupational groups, and was widely used in different countries worldwide (13, 14). Although vignette interviews can provide some information for researchers to understand the current state of people's MHL, it has some disadvantages. First, this method cannot produce a total or subscale score to directly reflect an individual's MHL level. MHL is a comprehensive concept with a rich meaning, including seven aspects. Although this method measures each of these seven aspects separately, the results cannot be combined into an overall MHL score due to the different forms of items. It is difficult to understand the MHL levels of people intuitively and specifically and make comparisons between individuals. Second, this measurement requires participants to identify a particular mental illness before answering follow-up questions. The participants' understanding of mental illness in the vignette determines their responses to subsequent questions; if someone does not identify the mental illness correctly, the subsequent answers may not reflect their MHL accurately (5). Third, because this method involves interviews, it has some common limitations of interviews, such as being time-consuming, laborious, etc.

The other method of MHL measurement is the scale-based measure. Researchers have developed several scales for MHL assessment. For instance, the Multiple-Choice Knowledge of Mental Illnesses Test (MC-KOMIT) developed by Compton et al. (15), comprising 33 items, was only used to measure the knowledge of serious and prevalent mental illnesses among lay samples and did not cover the attitude and stigma aspects of MHL. The Mental Health Knowledge Schedule developed by Evans-Lacko et al. (16), comprising 12 items, was designed to assess the general public's knowledge of mental health related to stigma, ignoring the identification, etiology, and other aspects of MHL. Compared with the vignette interview method, this method is suitable for large-scale tests and is time-efficient. The results are also more objective. However, neither of these measures can assess all seven attributes of MHL and report limited psychometric data (5).

For overcoming these limitations, O'Connor and Casey (17) from the Griffith University in Australia developed the Mental Health Literacy Scale (MHLS) to evaluate all the attributes of MHL. This scale contains 35 items designed to measure all seven domains of MHL conceptualized by Jorm. They tested the MHL in two diverse sets of individuals (372 university students and 43 mental health professionals) and found empirical support for its internal consistency, test-retest reliability, and construct validity. The result of the confirmatory factor analysis demonstrated a four-factor structure, but because of the low communalities and mean factor loadings (0.251), the authors suggested that a univariate structure was the most statistically and theoretically appropriate (17). The MHLS has proven to be an appropriate tool with good reliability and validity in different countries such as, Australia (18), United States (19), and New Zealand (20). In addition, the MHLS has been translated into Vietnamese (21) and Persian (22). The above researches have shown that MHLS has good reliability and validity and can be applicable to different populations.

Although the MHLS has been translated into Chinese (23, 24), its use has been limited. It was not developed for Chinese teachers in the context of primary and secondary school settings. Teachers are a distinct group, their MHL plays a vital role in the students' mental health. On one hand, teachers can provide psychological knowledge to students, on another hand, teachers with a high level of MHL can identify students who are in need of psychological help in time and make referrals for services. However, from the perspective of the current situation of education in China, the MHL of primary and secondary school teachers is not optimistic. Yu has pointed out that the number of mental health education teachers was insufficient in China, and they generally lacked of professional knowledge about mental health (25). Some researchers have proposed that improving the MHL level of primary and secondary school teachers is one of the favorable means to promote the development of MHL of students (26). In order to promote teachers' MHL, it is necessary to evaluate their MHL level effectively first. Therefore, it is very necessary to revise MHLS among Chinese teachers.

The criterion validity of the Chinese version of MHLS was also tested. Previous studies have found a significant positive association between MHL and health promoting behaviors (22), and a highly negative correlation between MHL and socially distancing oneself from those with mental illness (2, 27). Through a meta-analysis of 15 articles, Hadlaczky et al. (28) found that increased knowledge about mental health can increase support behaviors for people with mental illness and reduce stigma. Additionally, a robust literature was found supporting the role of MHL in improving mental health outcomes by reducing stigma among people with mental illness and facilitating help-seeking behavior and engaging with mental health care (7, 29). Accordingly, to test the criterion validity of the Chinese version of MHLS, we selected four validity criteria, including self-efficacy in coping with mental health problems, the mental health status, the perception of stigma associated with receiving mental health treatment, and the degree of avoidance of patients with mental illness. The hypotheses were as follows: Chinese version of MHLS would be positively related to self-efficacy in coping with mental health problems and mental health status. It would be negatively related to the perception of stigma associated with receiving mental health treatment and the degree of avoidance of patients with mental illness.

Accordingly, this study aimed to translate the MHLS into Chinese and examine the factor structure and psychometric properties of the Chinese version of the MHLS (MHLS-C) among Chinese teachers living in the Henan province. The more specific aims were as follows: (a) to explore the structural validity of the MHLS-C; (b) to determine the criterion validity of the MHLS-C by using the stigma associated with receiving mental health treatment, socially distancing oneself from someone with mental illness, self-efficacy in coping with mental health problems, and mental health related scales; (c) to examine the reliability of the MHLS-C; and (d) to explore the differences in the MHL of Chinese teachers between socio-demographic variables.

## Materials and Methods

### Participants

Participants were recruited at 4-day school mental health education workshops for primary and secondary school teachers in Henan province, China. We arrived at the workshop on the 1st day of training (December, 2019) and used their break time to distribute self-administered paper-and-pencil questionnaires. Each participant needed about 20 min to complete the questionnaire. A total of 433 questionnaires were distributed, and 367 questionnaires were returned and determined to be valid for the purpose of data analysis. The response rate was 84.8%. Participants in the present study were exclusively teachers. An informed consent form was presented on the first page of the survey citing the purposes and the voluntary nature of the survey, and this study protocol was approved by the Ethical Review Board of the Institution of Psychology and Behavior, Henan University.

### Measures

#### Demographic Variables

Participants completed a brief demographic questionnaire that included information regarding the teachers' sex, age, educational level, history of mental illness, and the subjects they taught.

#### Chinese Version of the Mental Health Literacy Scale

Mental health literacy was measured using the MHLS (17). The MHLS included 35 items. Participants rated each item using a four-point scale ranging from 1 (very unlikely true or very unhelpful) to 4 (very likely true or very helpful) (e.g., “To what extent do you think it is likely that personality disorders are a category of mental illness?” and “To what extent do you think it would be helpful for someone to improve their quality of sleep if they were having difficulties managing their emotions?”) or a five-point scale ranging from 1 (strongly disagree or definitely unwilling) to 5 (strongly agree or definitely willing) (e.g., “People with a mental illness could snap out if they wanted.” “How willing would you be to move next door to someone with a mental illness?”). The score of MHLS ranged from 35 to 160, with higher score implying an adequate MHL.

The translation of the MHLS to the Chinese language was achieved in the following steps. First, after obtaining permission from the author of the MHLS, a doctoral student and a post-doctor independently translated all 35 items of the MHLS into Chinese. They then discussed any discrepancies between their translation until they reached agreement. Second, A third bilingual doctoral graduate who had never seen the original MHLS back-translated the Chinese version to confirm the accuracy of the original translation. Then a panel comprised of one psychology professor, two psychology post-doctors and two psychology doctoral students viewed the original English version, the translated Chinese version, and the back-translated English version, discussed any discrepancies in different versions of the MHLS. With several modifications and wording revisions to fit the Chinese cultural context, the translated Chinese version of the MHLS (MHLS-C) was finalized.

#### Stigma Scale for Receiving Psychological Help

The SSRPH is a unidimensional scale. It consists of five items, and each question is rated from 0 (strongly disagree) to 3 (strongly agree), wherein higher scores indicated greater perception of stigma associated with receiving mental health treatment. In this study, it was used for exploring criterion validity. It demonstrated an adequate internal consistency of 0.72, and substantial convergence with negative attitudes toward mental health treatment (30). The reliability coefficient was 0.808 in this study.

#### Social Distance Scale

The Social Distance Scale that purports to measure the degree of avoidance of patients with mentally illness was developed by Link et al. (31). It contains seven items and each item is rated on a scale ranging from 1 (very unwilling) to 5 (very willing). All items are scored in reverse, with higher scores indicating a stronger desire for social distance (31). The reliability coefficient was 0.915 in this study.

#### The 12-item General Health Questionnaire (GHQ-12)

The GHQ-12 was developed by Goldberg to measure the mental health of teachers. We used it for exploring criterion validity in this study. This scale includes 12 items, with each item ranging from 0 to 3. All items were added to obtain the total score, making the score range 0–36, with a higher score indicating poorer mental health (32). The reliability coefficient was 0.812 in this study.

#### Self-Efficacy in Coping With Mental Health Problems

Participants were invited to rate the following items on a 10-point scale: “To what extent can you confidently identify people with mental illness correctly?”; “To what extent are you confident that you can get along well with people with mental illness?”; and “To what extent are you confident that you can offer effective advice and help to people with mental illness?” The end points of the scale were anchored (i.e., 1 = not at all confident, 10 = very confident), as was the midpoint (i.e., between 5 and 6, text read somewhat confident), but most points did not have a verbal description (33). We used this scale to explore criterion validity. The reliability coefficient was 0.871 in this study.

### Data Analyses

The evaluation of the MHLS-C items was a multi-step process that included the evaluation of (a) item analysis, (b) structural validity, (c) criterion validity, (d) reliability (internal consistency), and (e) socio-demographic difference.

Item analysis was calculated by using the Pearson's product moment correlations to correlate each item of the scale with the total score of the scale. These represent the extent to which items measure the same construct as the other items. Structural validity was established using a parallel analysis and the minimum average partial (MAP) method. The criterion validity of the scale was assessed using the Pearson's product moment correlation to correlate the MHLS-C with a number of different related measures (the SSRPH, Social Distance Scale, Self-efficacy in coping with mental health problems, and GHQ-12). We evaluated the internal consistency of the MHLS-C using Cronbach's α, which reflects the overall correlation between items within a scale. Moreover, socio-demographic differences in MHL were tested by using the independent sample *T*-test, analysis of variance, and correlation analysis.

Analyses were conducted using SPSS 25.0 and R 3.5.2 (with psych package, lavaan package, and GPArotation package). A significance level of alpha = 0.01 was used.

## Results

### Descriptive Statistics of Study Subjects

Of all the 367 participants, 10.4% were male (*n* = 38) and 89.6% were female (*n* = 329). Of all respondents, 93 (25.3%) were 20–29 years of age, 124 (33.8%) were 30–39 years of age, 127 (34.6%) were 40–49 years of age and 23 (6.3%) were 50 years or older; 312 (85.0%) had an undergraduate education or less, 55 (15.0%) had a master's degree or above; 118 (32.2%) had previous mental health problems or had friends with mental health problems and 249 (67.8%) had no experience with mental illnesses. A total of 123 respondents (33.5%) were full-time mental health teachers, 244 (66.5%) were teachers of other subjects.

The samples obtained from the Chinese teachers were utilized to generate a descriptive for the MHLS-C. Mean score for the scale was 115.35 (standard deviation = 10.63, Minimum = 86.00, Maximum = 147.00, 95% confidence interval = 114.26–116.44). Overall, the scale was somewhat normally distributed (Skewness = 0.200, Kurtosis = −0.197).

### Item Analysis

Most corrected item-total correlations were statistically significant, except the items 3, 5, 9, and 10 ranging from 0.040 to 0.597. There were 14 items lower than the recommended criterion of 0.300 (34). Ranking the MHLS-C total score in ascending order, the score of the top 27% was significantly lower than the score of the lowest 27% in most items (*p*s < 0.01, except item 3, 5, 9, 10). Referring to the results of the item analysis, the Cronbach's α after deleting the items (3, 5, 9, 10) not up to standard was 0.813. The results of the item analyses are located in a supplementary table (See Appendix A).

### Structural Validity

To investigate the factor structure, a parallel analysis and the minimum average partial (MAP) method were conducted on 35 items. The result of the parallel analysis suggested a five-factor structure, while the MAP method suggested a four-factor structure. Parallel analysis can overestimate the number of factors, while the MAP method can underestimate them (35). When parallel analysis and the MAP method recommend the same number, the result may be considered robust. If the numbers differ, factor analysis should be conducted beginning with the recommendations of the parallel analysis and decreasing the number of factors until the factor structure is interpretable, or by beginning with the recommendations of the MAP method and increasing the number of factors until the factor structure is interpretable.

Once a five-factor structure was set (principal component and Oblimin rotation), only three items loaded on the fourth factor, and three items loaded on the fifth factor, and the content of these items were all from “attitudes toward someone with a mental illness” factor in original questionnaire. When the four-factor structure was set (again with principal component and Oblimin rotation), the fourth and fifth factor in last model was combined into one. However, the loading of one item (number 15) was lower than 0.3. In order to stay consistent with prior studies and provide a better diagnostic criterion, item 15 was retained in the present scale. The results of factor analysis (with principal component and direct Oblimin rotation) are presented in Table 1.

**Table 1:** Factor analysis.

	**Factor1**	**Factor2**	**Factor3**	**Factor4**
	**(α = 0.760)**	**(α = 0.838)**	**(α = 0.499)**	**(α = 0.605)**
27. If I had a mental illness, I would not seek help from a mental health professional.	−0.726			
24. It is best to avoid people with a mental illness so that you don't develop this problem.	−0.628			
28. I believe treatment for a mental illness, provided by a mental health professional, would not be effective.	−0.618			
25. If I had a mental illness, I would not tell anyone.	−0.611			
26. Seeing a mental health professional means you are not strong enough to manage your own difficulties.	−0.600			
21. A mental illness is a sign of personal weakness.	−0.592			
23. People with a mental illness are dangerous.	−0.558			
22. A mental illness is not a real medical illness.	−0.524			
20. People with a mental illness could snap out if they wanted.	−0.396			
13. To what extent do you think it is likely that Cognitive Behavior Therapy (CBT) is a therapy based on challenging negative thoughts and increasing helpful behaviors?	−0.382			
15. To what extent do you think it is likely that the following is a condition that would allow a mental health professional to break confidentiality: if your problem is not life-threatening and they want to assist others to better support you?	−0.292			
31. How willing would you be to make friends with someone with a mental illness?		0.773		
33. How willing would you be to have someone with a mental illness marry into your family?		0.754		
32. How willing would you be to have someone with a mental illness start working closely with you on a job?		0.747		
29. How willing would you be to move next door to someone with a mental illness?		0.700		
35. How willing would you be to employ someone if you knew they had a mental illness?		0.695		
30. How willing would you be to spend an evening socializing with someone with a mental illness?		0.641		
34. How willing would you be to vote for a politician if you knew they had suffered a mental illness?		0.598		
11. To what extent do you think it would be helpful for someone to improve their quality of sleep if they were having difficulties managing their emotions?			0.546	
7. To what extent do you think it is likely that the diagnosis of Bipolar Disorder includes experiencing periods of elevated (i.e., high) and periods of depressed (i.e., low) mood?			0.535	
5. To what extent do you think it is likely that Dysthymia is a disorder?			0.519	
2. To what extent do you think it is likely they have generalized anxiety disorder?			0.460	
4. To what extent do you think it is likely that personality disorders are a category of mental illness?			0.448	
9. To what extent do you think it is likely that in general in China, women are MORE likely to experience a mental illness of any kind compared to men?			0.415	
10. To what extent do you think it is likely that in general, in Australia, men are MORE likely to experience an anxiety disorder compared to women?			0.406	
6. To what extent do you think it is likely that the diagnosis of Agoraphobia includes anxiety about situations where escape may be difficult or embarrassing?			0.379	
8. To what extent do you think it is likely that the diagnosis of Drug Dependence includes physical and psychological tolerance of the drug?			0.370	
3. To what extent do you think it is likely they have major depressive disorder?			0.353	
1. To what extent do you think it is likely they have Social Phobia?			0.351	
12. To what extent do you think it would be helpful for someone to avoid all activities or situations that made them feel anxious if they were having difficulties managing their emotions?			0.322	
16. I am confident that I know where to seek information about mental illness.				0.730
18. I am confident attending face to face appointments to seek information about mental illness.				0.701
17. I am confident using the computer or telephone to seek information about mental illness.				0.688
19. I am confident I have access to resources that I can use to seek information about mental illness.				0.658
14. To what extent do you think it is likely that the following is a condition that would allow a mental health professional to break confidentiality: If you are at immediate risk of harm to yourself or others?				0.411

In general, considering the content integrity and the consistency with prior study, the four-factor structure was accepted. However, the reliability coefficient of factors 3 (α = 0.499) and 4 (α = 0.605) was slightly lower, and the four-factor structure was inconvenient to provide a concise score of MHL in practice. To avoid these deficiencies, and maintain consistency with the original scale, we suggest that the univariate structure should be preferred.

### Criterion Validity

The mean and standard deviation for all the scales used for validity and the correlation coefficients between these scales can be seen in Table 2. The MHLS-C was positively related to self-efficacy in coping with mental health problems (*r* = 0.205, *p* < 0.01). The correlation coefficient between MHLS-C score and GHQ-12 score is −0.109 (*p* < 0.05), considering a higher score of GHQ-12 indicate poorer mental health status, so the MHL is positively correlated with mental health status. The MHLS-C was negatively related to the perception of stigma associated with receiving mental health treatment (*r* = −0.439, *p* < 0.01) and the act of socially distancing oneself from patients with mental illness (*r* = −0.549, *p* < 0.01). These results provide convincing evidence for the validity of the Chinese version of the MHLS among Chinese teachers.

**Table 2:** Intercorrelations between scales and reliabilities.

	**1**	**2**	**3**	**4**	**5**
(1) MHLS-C	1	−0.439^**^	−0.549^**^	0.205^**^	−0.109^*^
(2) SSRPH	−0.439^**^	1	0.142^**^	−0.132^*^	0.250^**^
(3) Social distance	−0.549^**^	0.142^**^	1	−0.220^**^	0.020
(4) Self-efficacy	0.205^**^	−0.132^*^	−0.220^**^	1	−0.299^**^
(5) GHQ-12	−0.109^*^	0.250^**^	0.020	−0.299^**^	1
Mean	115.35	2.81	24.26	17.84	18.55
Standard deviation	10.63	2.18	5.67	5.98	4.43
Range	86–147	0–9	8–35	3–30	12–40
α	0.792	0.808	0.915	0.871	0.812

*MHLS-C, Mental Health Literacy Scale-Chinese version; SSRPH, Stigma Scale for Receiving Psychological Help; GHQ-12, The 12-item General Health Questionnaire*.

* *p < 0.05*,

** *p < 0.01 (two-tailed), similarly hereinafter*.

### Reliability Analysis

The Cronbach's α coefficient of the MHLS-C was 0.792, demonstrating acceptable internal consistency. The internal consistency alpha values of the four factors were: 0.760 for factor 1, 0.838 for factor 2, 0.499 for factor 3, and 0.605 for factor 4. The reliability coefficient of factor 3 and factor 4 was slightly lower than the recommended criterion (34), but this is not surprising because factor 3 tested the understanding of different assessment and diagnostic tools and treatments, and factor 4 evaluated knowledge about different aspects of epidemiology of mental health and mental illness, both containing a multidimensional structure in nature, while internal consistency reliability intends to test the homogeneity of items. Despite this, both factors demonstrated acceptable internal consistency reliability. From another point of view, the original purpose of our research is to maintain the comprehensiveness of the measurement content and the simplicity of the results, we prefer a univariate structure, therefore, we are more concerned with the overall reliability of the scale.

### Socio-Demographic Differences in MHL

Socio-demographic variables (sex, education, experience of mental illness, and working as a full-time mental health teacher) are related to differences in MHLS-C score (Table 3). Regarding sex differences, females showed higher scores than males on the MHLS-C (*t* = −2.885, *p* < 0.01). Individuals with master's degrees or above showed a higher level of MHL than those with bachelor's degrees or below (*t* = −3.158, *p* < 0.01). Full-time mental health teachers scored significantly higher for MHL than teachers of other subjects (*t* = 4.583, *p* < 0.01). The results of Pearson's correlation analysis showed that the teachers' age was negatively related to the total score of MHL (*r* = −0.259, *p* < 0.01), demonstrating that younger teachers have a higher level of MHL.

**Table 3:** Socio-demographic difference in mental health literacy.

		**Mean ± Standard Deviation**	***t***
Sex	Male (*n* = 38)	110.68 ± 11.90	−2.885**
	Female (*n* = 329)	115.89 ± 10.36	
Education	Undergraduate or less (*n* = 312)	114.62 ± 10.37	−3.158**
	Master or above (*n* = 55)	119.47 ± 11.26	
History of mental illness	Yes (*n* = 118)	116.36 ± 10.43	1.261
	No (*n* = 249)	114.87 ± 10.71	
Full-time mental health teacher	Yes (*n* = 123)	118.84 ± 10.61	4.583**
	No (*n* = 244)	113.59 ± 10.22	

## Discussion

### General Discussion

The present study first examined the validity and reliability of the Chinese version of the 35-item MHLS among Chinese teachers. Parallel analysis showed that there were four factors in the MHLS-C, which was consistent with the original English version of the MHLS (17). In addition, the MHLS-C was significantly correlated with the self-efficacy for coping with mental health problems, mental health status, SSRPH, and socially distancing oneself from those with mental illness, indicating that the criterion validity of the MHLS-C was good. The Cronbach's α of MHLS-C total score and subscales was acceptable (0.499–0.838), indicating that the MHLS-C has good reliability. Therefore, the Chinese version of the 35-item MHLS has good validity and internal consistency reliability when applied to Chinese teacher groups. In the future, researchers can use this scale to effectively evaluate the level of mental health literacy of teachers in the context of Chinese culture.

The results of item analysis indicated that some of the items' discrimination were not satisfactory, in order to explore whether deleting these items would affect the quality of the scale, this study calculated the Cronbach's α after deleting these items. The Cronbach's α changed from 0.792 to 0.813 after deletion. The change in coefficient is very slight, and considered of the differences related to the sample composition, cultural and other factors. In order to ensure the integrity of the structure of the original scale, it is finally decided not to delete the above items and keep the 35 items of the original scale unchanged. The 35 items of the scale ensure that it can measure all seven attributes of the MHL.

The parallel analysis showed that there were four factors in the MHLS-C, which was in accordance with the results of the original research. Authors of MHLS focused on the comprehensiveness and simplicity of the measurement results, so abandoned the optimal statistical factor structure and preferred the univariate structure of the scale (17). For the same reason, our study also supported the univariate structure of MHLS-C. However, whether the scale is a univariate structure needs to be further tested, we will collect more data and conduct further tests on the scale structure. Another Chinese version of MHLS showed eight factors, and after naming and combining the same factors, the researcher ended up with six factors (23). This inconsistency may have been due to the difference in methods used to extract the factors. We used the parallel analysis and MAP, same as that used by the author of MHLS original version, while the other Chinese researcher adopted the eigenvalue >1.0 rule. This method was affected by the number of observed variables, which may lead to excessive extraction of the factors (36). Although the results of this study echoed the original study's univariate structure, due to the limited sample of subjects and other reasons, no further statistical analysis was conducted on the factor structure of the scale. Therefore, we call for more researchers to investigate its factor structure in the future.

The total internal consistency α coefficient of the MHLS-C is 0.792, which is slightly lower than the original version of MHLS (α = 0.87). Several competing possibilities are plausible for this discrepancy. The first reason could be poor translation of items; although the translation process was fairly rigorous, arguing against this possibility, but not excluding it entirely. Poorly translated items could induce spurious responses that would weaken the association of the items with the intended factor. A more likely explanation is conceptual ambiguity. Items may have been interpreted in a manner inconsistent with their original meaning. It is a common occurrence when translating English scales into Chinese (37). In addition, the internal consistency α coefficient of MHLS-C is close to two other Chinese versions of MHLS (α = 0.81, α = 0.704), the Vietnamese version (α = 0.72), and the Persian version (α = 0.74). These results indicate that the MHLS has a good internal consistency and stability when used with different groups.

The current study explored the impact of socio-demographic variables on the levels of MHL. Differences were found regarding sex, age, educational level, and whether the participants were working as full-time mental health teachers. In general, females, younger teachers, teachers with higher educational level, and full-time mental health teachers showed higher levels of MHL, in line with previous studies. For example, researchers have found that males show lower MHL levels, less favorable attitudes toward mental health, higher stigma concerns, and lower intentions to seek help (38, 39). Fisher and Goldney (40) have pointed out that age was one of the factors influencing the MHL of teachers, and young teachers were better at identifying mental illness than older teachers. At the same time, they were more willing to seek professional psychotherapy. Gorczynski et al. (41) found that postgraduate students exhibited higher levels of MHL than undergraduate students. A study conducted in Iran also found that participants with low education levels had a lower mean MHL score (22). These relatively consistent results indicated that the MHL of teachers with different characteristics is different. Therefore, in further intervention studies, researchers should fully consider these differences and carry out targeted intervention to improve their MHL.

### Limitations and Future Research

This study has some limitations. First, although we have adopted strict translation procedures in this study, the influence of cultural background differences cannot be avoided in translation. Studies have shown that due to the influence of Confucianism, Chinese people have higher stigma against mental diseases compared with people in other countries (42). Since the original scale was compiled under the cultural background of Australia, the Chinese people's understanding of the items will inevitably be influenced by the traditional Chinese culture, thus reducing the applicability of the scale under the Chinese culture. Second, the sample range of this study is relatively limited. The subjects in this study were primary and secondary school teachers in Henan Province, China, so we should be cautious in generalizing the conclusions of this study. Third, the present study did not determine test-retest reliability, so the model stability needs to be further tested.

Future Research should be based on the Chinese cultural background, using qualitative research methods to develop a set of mental health literacy scale. And future studies should attempt to recruit more diverse samples. On the one hand, the categories of sample groups should be increased, such as psychiatrists, psychologists, civil servants, ordinary people, etc. In this way, researchers can not only test the population applicability, but also determine the discriminative validity of this scale. On the other hand, the distribution range of samples should be expanded to verify the applicability of the scale in China. In addition, future studies should increase the number of samples, supplement the retest reliability of MHLS-C, and conduct confirmatory factor analysis on the basis of the factor structure results of this study.

## Conclusion

The present study developed the Chinese version of the MHLS, and further examined its validity and reliability. The results showed that it is a useful instrument for assessment of the MHL among Chinese teachers. This scale may be used to identify the MHL level of teachers, identifying their knowledge gaps and erroneous beliefs concerning mental health issues, accordingly providing them adequate mental health education, and ultimately promoting their and their students' mental health. In addition, this tool can also evaluate the effectiveness of mental health education programs. The use of the MHLS-C can also allow comparisons between Chinese teachers and other teacher groups in different countries using the three other-language versions of the MHLS (English, Persian, and Vietnamese). In short, the present study provides much promise for improving the MHL of Chinese teachers and promoting the development and perfection of mental health education in China.

## Data Availability Statement

The raw data supporting the conclusions of this article will be made available by the authors, without undue reservation.

## Ethics Statement

The studies involving human participants were reviewed and approved by Ethical Review Board of the Institution of Psychology and Behavior, Henan University. The patients/participants provided their written informed consent to participate in this study.

## Author Contributions

YL was the principal investigator for the study, generated the idea and designed the study, was the primary writer of the manuscript, and approved all changes. SC and WW supported the data input, data analysis, and writing up the manuscript. KC and SW supported the data collection. All authors were involved in developing, editing, reviewing, providing feedback for this manuscript, and have given approval of the final version to be published.

## Conflict of Interest

The authors declare that the research was conducted in the absence of any commercial or financial relationships that could be construed as a potential conflict of interest.

## Abbreviations

MHL: Mental health literacy
MHLS: Mental Health Literacy Scale
HL: Health Literacy
MC-KOMIT: Multiple-Choice Knowledge of Mental Illnesses Test
MHLS-C: Chinese version of the MHLS
SSRPH: Stigma Scale for Receiving Psychological Help
GHQ-12: 12-item General Health Questionnaire
MAP: minimum average partial
CBT: Cognitive Behavior Therapy.
