# Supplementary material for: Initial Validation of a Chinese Version of the Mental Health Literacy Scale Among Chinese Teachers in Henan Province
Source: Front Psychiatry. 2021 Jun 9;12:661903. doi: 10.3389/fpsyt.2021.661903 (PMC8219938; doi:10.3389/fpsyt.2021.661903)
Supplement: Supplementary file 1 [file Table_1.docx]

Appendix A：

Table S1 The results of item analysis (*n*=367).

| Item | Corrected item-total correlation | Cronbach’s *α* if item-deleted | *t* |
| --- | --- | --- | --- |
| 1 | .216^**^ | .791 | -4.149^**^ |
| 2 | .274^**^ | .789 | -5.620^**^ |
| 3 | .040 | .797 | -.802 |
| 4 | .235^**^ | .792 | -4.049^**^ |
| 5 | .087 | .797 | -1.764 |
| 6 | .176^**^ | .793 | -3.311^**^ |
| 7 | .442^**^ | .784 | -8.883^**^ |
| 8 | .183^**^ | .793 | -3.080^**^ |
| 9 | .074 | .798 | -1.684 |
| 10 | .078 | .797 | -1.619 |
| 11 | .410^**^ | .785 | -7.815^**^ |
| 12 | .278^**^ | .790 | -6.304^**^ |
| 13 | .376^**^ | .787 | -6.879^**^ |
| 14 | .215^**^ | .791 | -3.020^**^ |
| 15 | .291^**^ | .789 | -4.457^**^ |
| 16 | .357^**^ | .787 | -6.204^**^ |
| 17 | .264^**^ | .790 | -4.458^**^ |
| 18 | .327^**^ | .788 | -5.560^**^ |
| 19 | .397^**^ | .785 | -6.840^**^ |
| 20 | .201^**^ | .794 | -3.288^**^ |
| 21 | .417^**^ | .785 | -7.687^**^ |
| 22 | .413^**^ | .785 | -7.003^**^ |
| 23 | .433^**^ | .784 | -8.249^**^ |
| 24 | .524^**^ | .780 | -10.862^**^ |
| 25 | .471^**^ | .782 | -9.033^**^ |
| 26 | .465^**^ | .783 | -8.069^**^ |
| 27 | .506^**^ | .781 | -9.301^**^ |
| 28 | .450^**^ | .784 | -9.143^**^ |
| 29 | .516^**^ | .780 | -9.200^**^ |
| 30 | .597^**^ | .776 | -12.702^**^ |
| 31 | .560^**^ | .777 | -11.104^**^ |
| 32 | .523^**^ | .779 | -10.736^**^ |
| 33 | .462^**^ | .782 | -7.838^**^ |
| 34 | .362^**^ | .788 | -7.282^**^ |
| 35 | .477^**^ | .782 | -9.461^**^ |

Note^: **^p<.01 (two-tailed)
